# The ideal time of systemic metronidazole and amoxicillin administration in the treatment of severe periodontitis: study protocol for a randomized controlled trial

**DOI:** 10.1186/s13063-018-2540-8

**Published:** 2018-03-27

**Authors:** Magda Feres, Belén Retamal-Valdes, Maria Josefa Mestnik, Luciene Cristina de Figueiredo, Marcelo Faveri, Poliana M. Duarte, Aretuza Fritoli, Elisangela Faustino, Maria Luisa Silveira Souto, Michelle de Franco Rodrigues, Marcela Giudicissi, Bárbara Campos Lara Nogueira, Luciana Saraiva, Giuseppe Alexandre Romito, Cláudio Mendes Pannuti

**Affiliations:** 1 Department of Periodontology, Dental Research Division, Guarulhos University, Guarulhos, São Paulo Brazil; 2 Division of Periodontics, Department of Stomatology, School of Dentistry, University of Sao Paulo, São Paulo, Brazil

**Keywords:** Periodontitis, Periodontal Disease, Metronidazole, Amoxicillin, Scaling and Root Planing, Treatment, Antibiotics

## Abstract

**Background:**

The combination of systemic metronidazole (MTZ) and amoxicillin (AMX) with scaling and root planing (SRP) has shown to be an effective periodontal treatment. However, some essential issues associated with the use of these antibiotics remain unanswered, such as the ideal time of administration during the course of periodontal treatment. Although these agents are often prescribed after the healing phase of the SRP procedure, there is biological plausibility to support its use in conjunction with the mechanical treatment. However, to date, no placebo controlled randomized clinical trial (RCT) has directly compared these two protocols. Therefore, the aim of this RCT is to compare the clinical, microbiological and immunological effects of the adjunctive systemic MTZ + AMX administered in different phases of the treatment of severe periodontitis.

**Methods:**

Subjects with severe periodontitis (*n* = 180) are being randomly assigned into three groups (*n* = 60/group): (i) SRP-only (control group), SRP in combination with 400 mg MTZ + 500 mg AMX, starting (ii) at the first SRP session (active phase group), or (iii) after 3 months of its completion (healing phase group). All volunteers are receiving clinical and microbiological evaluation at baseline, 3, 6 and 12 months, and immunological assessment at baseline and 12 months post-therapy. Nine subgingival biofilm samples are being collected per subject and analyzed for counts and proportions of 40 bacterial species by checkerboard DNA-DNA hybridization, and six gingival crevicular fluid samples are being collected and analyzed for the levels of 20 chemokines by multiplex immunoassay. The primary outcome variable is the number of volunteers reaching the clinical endpoint for treatment (≤ 4 sites with probing depth ≥5 mm) at 1 year post-therapy. Differences in clinical, microbiological and immunological parameters among groups and over time will be evaluated using analysis of variance, analysis of covariance and the Chi-square and Tukey tests. Microbiological and immunological analyses will be performed using adjustments for multiple comparisons. Statistical significance will be set at 5%.

**Trial registration:**

ClinicalTrials.gov, NCT02954393. Registered on 3 November 2016.

**Electronic supplementary material:**

The online version of this article (10.1186/s13063-018-2540-8) contains supplementary material, which is available to authorized users.

## Background

Periodontitis is an infectious-inflammatory disease triggered by oral microorganisms organized in biofilms, resulting in loss of periodontal bone support and, in many cases, in tooth loss [1, 2]. The standard care for periodontitis consists of oral hygiene instruction (OHI) and mechanical debridement of the root surfaces (i.e., scaling and root planing (SRP)), aiming to remove calculus and biofilm. Although this is a very effective approach in many cases [3], it has inherent limitations, especially in patients with advanced disease. As a result, SRP may be not sufficient to change the bacterial profile associated with periodontitis to a profile compatible with periodontal health [4]. For this reason, other treatments, such as adjunctive systemic antibiotics, have been advocated.

There is strong evidence to support the use of systemic antibiotics as adjuncts to SRP in the treatment of severe periodontitis [4–9]. Over the course of recent decades, a diverse range of antimicrobials has been used as adjuncts to nonsurgical periodontal treatment. Among these, the association of metronidazole (MTZ) and amoxicillin (AMX) has shown to be effective in the treatment of severe periodontitis in adults [5, 10–14].

However, in spite of the evidence supporting the efficacy of adjunctive systemic antimicrobials, there is a lack of evidence to support well-defined clinical protocols [5] and some questions remain unanswered, such as: “Which is the best timing for the administration of the antibiotics, during the active phase of therapy or on re-evaluation (3 months after active treatment)?”

Some biologic concepts support the use of antibiotics during the active phase of therapy [5], together with SRP treatment. Previous authors have suggested that mild and sequential disturbances of the biofilm structure may not be sufficient to alter its highly stable climax community. On the other hand, rapid and striking reductions of the subgingival microbiota could result in a more beneficial recolonization of the periodontal pockets [15, 16]. Thus, the association between SRP and systemic antibiotics during the initial therapy would have a greater potential to create an entirely new and stable climax community, similar to that observed in health. In addition, since recolonization is normally achieved at 3 months after SRP, there is a possibility that antimicrobials administered at re-evaluation would work almost as a maintenance scaling. Furthermore, if antibiotics are given during the active phase of the therapy, periodontal tissue inflammation will be more intense, which would allow a higher concentration of antibiotic delivered to the subgingival area, as a result of the increased levels of gingival crevicular fluid (GCF) and greater permeability of capillaries, leading to greater antibiotic uptake [17].

Despite the biological plausibility associated with antibiotic intake in the active phase of periodontal treatment, clinicians tend to postpone the decision whether to use these agents to the re-evaluation phase. Therefore, in daily clinical practice, antibiotics are more likely to be used at 3–6 months after mechanical treatment.

So far, only two investigations - one retrospective study [17] and a randomized clinical trial (RCT) on aggressive periodontitis [18] - have investigated the best time for the administration of systemic antibiotics. The results of both studies suggest greater clinical benefits when MTZ and AMX were prescribed at the initial phase of therapy. It should be noticed, however, that the study of Griffiths et al. [18] was not designed to compare the effectiveness of the antibiotics given in different phases of treatment. The study was designed to test MTZ + AMX in the active phase and the control group took the agents after 6 months. Thus, up to now, no RCT has directly addressed the question of which is the best time for the administration of antibiotics in patients with severe periodontitis.

## Methods/design

### Aim, design and setting of the study

The aim of this study is to compare the effectiveness of the two following protocols of MTZ plus AMX, used as adjuncts to SRP in the treatment of severe periodontitis: (i) MTZ + AMX administered at the active phase of the periodontal treatment (together with SRP) or (ii) after the healing phase (3 months after SRP). In order to address this aim, we designed a double-blinded, three-armed, placebo-controlled and bi-centric RCT, which is being conducted at Guarulhos University (UNG; Guarulhos, SP, Brazil) and University of São Paulo (USP-SP; São Paulo, SP, Brazil).

The protocol was elaborated according to the Standard Protocol Items: Recommendations for Interventional Studies (SPIRIT) guidelines and using the SPIRIT checklist (Additional file 1) [19], and was registered at ClinicalTrials.gov (NCT02954393). The study acronym (M.O.M.E.N.T) stands for “Amoxicillin and Metronidazole Before or After Mechanical Periodontal Treatment”.

### Ethical considerations

This trial is being conducted according to the principles of the Declaration of Helsinki for studies in humans. The protocol was approved by the Institutional Review Board of UNG (Clinical Research Ethics Committee, CAAE: 32.465.714.4.1001.5506) and USP-SP (USP Ethics Committee, CAAE: 32.465.714.4.2001.0075). All eligible volunteers are informed about the nature, potential risks and benefits of their participation in this study and sign an informed consent form.

### Subject population and inclusion/exclusion criteria

Systemically healthy volunteers with untreated severe periodontitis are being selected from the Center for Clinical Trials of UNG and Periodontal Clinic of USP-SP. Subjects are being selected according to the following inclusion criteria: ≥ 35 years of age, presence of at least 15 teeth (excluding third molars and teeth with advanced decay referred for extraction), a minimum of 6 teeth with at least one site each with probing depth (PD) and clinical attachment level (CAL) ≥5 mm and at least 30% of the sites with PD and CAL ≥4 mm (mm) and bleeding on probing (BOP). Exclusion criteria are as follows: pregnancy, breastfeeding, current smokers and former smokers within the past 5 years, systemic diseases that could affect the progression of periodontitis (e.g. diabetes mellitus, immunological disorders, osteoporosis), SRP or antibiotic therapy in the previous 6 months, long-term intake of anti-inflammatory medications, need for antibiotic pre-medication for routine dental therapy, use of orthodontic appliances, extensive prosthetic rehabilitation and allergy to MTZ and/or AMX.

### Interventions

**Fig. 1:**
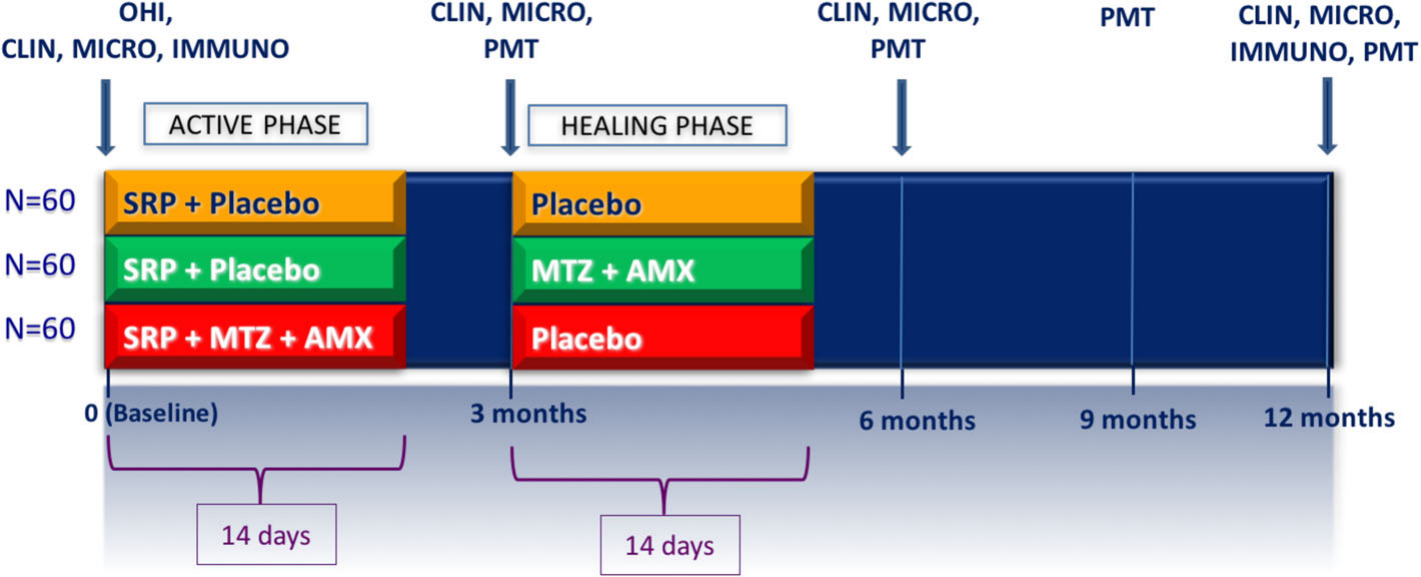
Experimental design of the M.O.M.E.N.T. study. CLIN: clinical assessment; MICRO: microbiological assessment; IMMUNO: immunological assessment; OHI: oral hygiene instruction; SRP: scaling and root planing; MTZ: metronidazole (400 mg/thrice daily); AMX: amoxicillin (500 mg/TID); PMT: periodontal maintenance therapy

At baseline, all volunteers fulfill a structured questionnaire comprising information about demographic, oral and general health data. Subsequently, they are submitted to a complete periodontal clinical assessment, collection of subgingival biofilm and GCF samples, OHI, supragengival scaling with ultrasonic scaler (Cavitron® Select™ Ultrason Scaler, Denstply, New York, USA) and curettes (Millenium, GOLGRAN, *São Caetano do Sul*, SP, Brazil). Subsequently, each volunteer is randomly allocated to one of the following therapeutic groups: (i) control (*n* = 60) - SRP + placebo concomitant to SRP and at 3 months post-SRP; (ii) active phase group (n = 60) - SRP + AMX (500 mg) + MTZ (400 mg) concomitant to SRP and placebo at 3 months post-SRP; and (iii) healing phase group (n = 60) - SRP + placebo concomitant to SRP and AMX(500 mg) + MTZ(400 mg) at 3 months post-SRP (Fig. 1).

All medications and placebos are given thrice a day (TID) for 14 days and placebo medications include two tablets, one of MTZ and one of AMX. In the active phase of treatment, the medications (antibiotics or placebos) start immediately after the first SRP session. At 3 months post-therapy (after the healing phase) the medications start immediately after the maintenance appointment.

Antibiotics and placebos are being specially prepared for this study by the same pharmacy (*Gallen Pharmacy and Manipulation Ltda*, Maringá, PR, Brazil). All tablets have the same color and size, and are stored in properly coded opaque plastic bottles, with 22 units. Volunteers, personnel (researchers responsible for the treatment) and the examiners are blind to the intervention.

Volunteers use 21 tablets in the first week and 21 in the second week. They are not informed about the total number of tablets in each bottle (22 capsules). They return the bottles at the second week of treatment and receive new ones containing the same amount of medication for the second week. Thus, it is possible to count the number of capsules ingested and the residual capsule (22nd) left in the bottle, in order to monitor compliance. In addition, they are also monitored every 2 days, personally (during the SRP treatment) or via telephone. At the end of the medication period (14th day), in the active phase and in the healing phase, volunteers respond to a questionnaire about any self-perceived side effects.

SRP is performed using Gracey curettes (conventional and mini-fives) numbers 5/6, 7/8, 11/12 and 13/14 under local anesthesia, and the treatment is completed in four to six sessions of approximately 1 h, distributed over a period of 14 days. At the end of each session, the clinical coordinator of each center evaluates the effectiveness of SRP using the outcome “smoothness of the scaled roots”. All volunteers receive periodontal maintenance therapy every 3 months post-treatment until the end of the study (12 months post-therapy), including OHI and prophylaxis with ultrasonic scaler and curettes.

### Clinical examination

Two calibrated examiners (B.R.V. and L.S.), one from each center (UNG and USP-SP), perform the clinical evaluations and sample collection. The following parameters are evaluated during clinical examination: visible plaque [20], gingival bleeding (0/1), BOP (0/1), suppuration (0/1), PD (mm) and CAL (mm) at six sites per tooth (mesiobuccal, buccal, distobuccal, distolingual/palatal, lingual/palatal and mesiolingual/palatal) in all teeth, excluding third molars. PD and CAL measurements are rounded to the nearest millimeter using a North Carolina periodontal probe (Hu-Friedy, Chicago, IL, USA).

The two examiners were trained and calibrated prior to and during the trial, in order to achieve maximum reproducibility in the measurements. The methodology used for the inter-examiner and intra-examiner calibration was recommended by Araujo et al. [21], where the standard error of measurement for continuous periodontal clinical parameters (PD and CAL) is evaluated. For the other clinical variables, the average level of agreement between the examiners is determined and considered satisfactory when greater than 90% (Kappa test).

Clinical measurements and microbiological assessment are performed at baseline, 3, 6 and 12 months post-therapy. Immunological assessment is conducted at baseline and 12 months post-therapy.

### Primary and secondary outcome variables

The primary outcome variable is the number of volunteers reaching the clinical endpoint for treatment (≤ 4 sites with PD ≥5 mm) at 1 year post-therapy [12, 14].

Secondary outcome variables are: difference between baseline and 12 months post-therapy for CAL gain and PD reduction (in the full mouth and in different PD categories), mean number and percentage of moderate (PD = 4–6 mm) and deep (PD ≥7 mm) sites at 12 months; number and percentage of volunteers with 0, 1–2 or ≥ 3 sites with PD ≥6 mm at 12 months post-therapy; number of sites with PD ≥5 mm or ≥6 mm at all post-treatment time points; differences in the occurrence of adverse events among therapeutic groups; differences in the counts and proportions of 40 bacterial species and in the counts of 20 chemokines in the GCF at all post-treatment time points.

### Sample size calculation

Sample size calculation was based on the primary outcome (number of volunteers reaching the clinical endpoint of ≤4 sites with PD ≥ 5 mm at 1 year post-therapy). Considering a difference of 31 percentage points between groups (31% vs 62%) as regards the primary outcome [14], a significance level of 5%, and 90% power, 50 subjects per group would be necessary. Considering a 20% rate of loss to follow up, it would be necessary to include 60 volunteers per group (total 180 subjects).

### Randomization

An investigator (C.M.P.), not involved in the inclusion and treatment of the patients, assigned the study participants to one of the three treatment groups by means of a computer-generated random sequence (Random Allocation Software, http://random-allocation-software.software.informer.com). Randomization is stratified by center with the use of permuted blocks of 3, 6 and 9. The study coordinator organizes the bottles with the tablets in opaque plastic bags labeled with the volunteer number. The bags are handed directly to the volunteer by the study coordinator in the active and healing phases. This sequence of procedures assures allocation concealment.

### Microbiological monitoring (baseline, 3, 6 and 12 months)

#### Sample collection

Nine subgingival samples are being collected per volunteer, three in each of the following categories: shallow (PD ≤3 mm), moderate (PD = 4–6 mm) and deep (PD ≥7 mm). The selected sites are located on non-contiguous interproximal dental surfaces and preferably distributed across the four quadrants. Sites located in teeth with poorly adapted prostheses, furcation lesion, extensive caries and/or endo-periodontal lesion are not selected. After the clinical assessment, the supragingival biofilm is removed and the subgingival sample is collected with individual sterile number 11/12 mini-five Gracey curettes, positioned at the most apical portion of the sites and with a single scaling movement in the apico-coronal orientation. The samples are immediately placed in separate Eppendorf tubes containing 150 μL of TE buffer solution (10 mM Tris-HCL, 1 mM EDTA, pH 7.6) and then 100 μL of 0.5 M NaOH is added to each tube. The samples are stored at − 20 °C. The tubes containing the samples are previously identified with the volunteer’s code, date and site collected.

#### Processing of microbiological samples

The counts and proportions of 40 bacterial species will be determined in each individual sample using a modification [22] of the checkerboard DNA-DNA hybridization technique [23]. The microbiological analysis will be entirely performed at the Molecular Biology Laboratory of UNG. In brief, the suspensions containing the bacterial biofilm samples collected are boiled for 10 min and neutralized by the addition of 0.8 mL of 5 M ammonium acetate, and the DNA is released in the solution. The released DNA will be then placed into the extended slots of Minislot 30 apparatus (Immunetics, Cambridge, MA, USA) concentrated on a 15 × 15 cm positively charged nylon membrane (Boehringer Mannheim, Indianapolis, IN, USA) and fixed to the membrane by boiling it at 120°C for 20 min. Subsequently, the membrane is placed in a Miniblotter 45 (Immunetics, Cambridge, MA, USA) with the lanes of DNA at 90 ° to the lanes of the device. Digoxigenin-labeled whole genomic DNA probes for 40 bacterial species are hybridized in individual lanes of the Miniblotter. After hybridization, the membrane is washed in highly astringent solution and the DNA probes are detected using the antibody to digoxigenin conjugated with alkaline phosphatase, and chemiluminescence detection. The last two lanes in each run contain standards at concentrations of 10^5^ and 10^6^ cells of each species. Signals will be evaluated visually by comparison with the standards for the test species on the same membrane by a calibrated examiner (*k* test = 93%). The sensitivity of this assay will be adjusted to allow detection of 10^4^ cells of a given species by adjusting the concentration of each DNA probe [22–24]. The mean counts (10^5^ cells) of individual bacterial species will be averaged within each subject and then across subjects in each group. The percentage of the total DNA probe counts will be determined initially in each site, then per subject, and averaged across subjects in the three groups at each time point. The sum of the individual mean proportion will be computed for each microbial complex described by Socransky et al. [25].

### Immunological monitoring (baseline and 12 months)

#### Sample collection

Six GCF samples are selected, two in each of the following categories: shallow (PD ≤3 mm), intermediate (PD = 4–6 mm) and deep (PD ≥7 mm). The selected sites are located on non-contiguous interproximal dental faces and preferably distributed among the four quadrants. After removal of the supragingival biofilm with sterile cotton pellets, the sites are isolated with cotton rolls and gently dried with an air syringe to eliminate the possibility of contamination with saliva. GCF is collected by inserting standard paper strips (Periopaper, Oraflow Inc., Smithtown, NY, USA), approximately 2 mm into the periodontal pocket for 20 s. Strips visually contaminated with blood are discarded. The GCF volume is measured in a calibrated device (Periotron 8000, Proflow Inc., Amityville, NY, USA) and the readings are converted to an actual volume (microliter, μL) by reference to a standard curve. Strips from the six selected sites are immediately placed into separate microcentrifuge tubes and stored at − 80 °C for subsequent assays.

#### Processing of samples

The GCF samples will be evaluated for their content of 20 cytokines by Multiplex Bead Immunoassay (MAGPIX® System, Merck Millipore, Billerica, MA, USA): 75 uL of PBS will be placed in each microtube containing the individual samples and then they will be vortexed for 15 s and centrifuged for 5 min at 1500 × g for elution. Samples will be analyzed using a multi-analyte method by means of a 20-multiplex fluorescent bead-based immunoassay for 20 cytokines/chemokines (granulocyte macrophage-colony stimulating factor (GM-CSF), interferon-γ (INF-γ), IL-1β, IL-2, IL-4, IL-5, IL-6, IL-7, IL-8, IL-10, IL-12 (p70), IL-13, IL-17A, IL-21, IL-23, CXCL11 (ITAC), macrophage inflammatory protein (MIP)-1α, MIP-1β, MIP-3α, TNF-α) using commercially available kits (MILLIPLEX® MAP 384-Well High Sensitivity Human Cytokine Magnetic Bead Panel, EMD Millipore, Billerica, MA, USA) and a plate reader (MAGPIX® System, Merck Millipore, Billerica, MA, USA), according to the manufacturer’s recommendations. The amount of protein in each sample will be extrapolated from standards using appropriate software (Beadview EMD Millipore, Billerica, MA, USA). The minimum detectable concentration of GM-CSF, IFN-γ, IL-1β, IL-2, IL-4, IL-5, IL-6, IL-7, IL-8, IL-10, IL-12 (p70), IL-13, IL-17A, IL-21, IL-23, ITAC, MIP-1α, MIP-1β, MIP-3α, TNF-α are 0.86 pg/ml, 0.37 pg/ml, 0.32 pg/ml, 0.26 pg/ml, 1.49 pg/ml, 0.31 pg/ml, 0.14 pg/ml, 0.38 pg/ml, 0.16 pg/ml, 0.98 pg/ml, 0.27 pg/ml, 0.16 pg/ml, 0.57 pg/ml, 0.19 pg/ml, 6.91 pg/ml, 1.06 pg/ml, 1.86 pg/ml, 0.71 pg/ml, 0.70 pg/ml and 0,24 pg/ml, respectively. The results will be reported as concentrations of cytokines/chemokines per volume of GCF (pg/μL).

### Data collection and management

Clinical data are entered directly onto electronic spreadsheets (Microsoft® Excel 2011®, version 14.4.8, Copyright © 1990, Microsoft, Santa Rosa, California, USA), at the time of clinical examination, by a single investigator in each center. Data quality will be validated by checking missing data, out-of-range values and invalid responses.

### Statistical analysis

#### Clinical monitoring

The mean percentage of sites with visible plaque, gingival bleeding, BOP, suppuration, number of sites with PD ≥5 mm and PD ≥6 mm, and mean PD and CAL are computed for each subject and then averaged across subjects in each group. Generalized estimating equation (GEE) tests will be used to evaluate the differences within each group, among groups and in experimental times. The data on reduction of the number of sites with PD ≥5 mm and PD ≥6 mm and on PD reduction and CAL gain in full-mouth assessment and initially intermediate (PD = 4–6 mm) and deep (PD ≥7 mm) sites will be evaluated by multilevel analysis using the GEE test. The chi-square test will be used to compare differences in the frequency of the gender of patients exhibiting different categories of residual sites in post-therapy experimental times (low, moderate or high risk of disease progression) according to Feres et al. [12] and Borges et al. [14], and of self-perceived adverse effects. The level of significance will be set at 5%. The data will be evaluated using intention-to-treat analysis with last observation carried forward.

#### Microbiological monitoring

Microbiological data will be expressed in counts (levels) and proportion counts of DNA probes. The data will be expressed as counts × 10^5^ at each site by volunteers and then by volunteers within each group at each study time. The same way, the proportion and prevalence of each species will be computed for each site, then the means between the sites in each volunteer will be calculated, and then the volunteers of the same therapeutic group will be calculated at each experimental time. The differences among the groups and between the experimental times will be evaluated by multi-level analysis using the GEE test. Adjustments for multiple comparisons [26] will be performed when the 40 bacterial species will be evaluated simultaneously.

#### Immunological monitoring

The concentration, total count and proportion of each cytokine/chemokine in the GCF will be assessed per volunteer and then per volunteer within each group at each study time. Similarly, the proportion will be computed for each site, then the means among the sites in each volunteer will be calculated, and then the volunteers of the same therapeutic group will be calculated in each experimental time. The differences among the groups and within each group, among the experimental times will be evaluated by multi-level analysis using GEE. All analyses will be adjusted for multiple comparisons [25].

## Trial status

This is an ongoing trial. Both centers are still recruiting study subjects. At the time of submission of this manuscript, 73 participants and 55 participants had been included at the UNG and USP-SP centers, respectively.

## Additional file

Additional file 1: SPIRIT checklist. (DOC 123 kb)

## Abbreviations

AMX: Amoxicillin
BOP: Bleeding on Probing
CAL: Clinical Attachment Level
DNA: DeoxyriboNucleic Acid
GCF: Gingival Crevicular Fluid
GEE: Generalized Estimating Equations
IL: Interleukin
M: Molar concentration
min: Minutes
ml: Milliliters
mm: Millimeters
MTZ: Metronidazole
OHI: Oral Hygiene Instruction
PBS: Phosphate-Buffered Saline
PD: Probing Depth
RCT: Randomized Clinical Trial
SPIRIT: Standard Protocol Items: Recommendations for Interventional Studies
SRP: Scaling and Root Planing
TID: Thrice A Day
TNF: Tumor Necrosis Factor
uL: Microliters
UNG: Guarulhos University
USP-SP: University of São Paulo
